# Acute hepatitis with portal and mesenteric vein thrombosis revealing SARS-CoV-2 infection: Case report and literature review

**DOI:** 10.1016/j.amsu.2022.103706

**Published:** 2022-05-02

**Authors:** Abdelhakim Harouachi, Tariq Bouhout, Hanane Hadj Kacem, Badr Serji, Hayat Berkhli, Hamid Madani, Tijani EL Harroudi

**Affiliations:** a Surgical Oncology Department, Regional Oncology Center, Mohammed VI University Hospital, Oujda, Morocco; b Mohammed First University Oujda, Faculty of Medicine and Pharmacy Oujda, Oujda, Morocco; c Department of Intensive Care Unit, Regional Oncology Center, Mohammed VI University Hospital, Oujda, Morocco; d Department of Radiology, Regional Oncology Center, Mohammed VI University Hospital, Oujda, Morocco

**Keywords:** Acute hepatitis, Portal and mesenteric vein thrombosis, COVID-19

## Abstract

Novel coronavirus disease 2019 (COVID-19) is a single-stranded RNA virus identified for the first time in Wuhan, China, and it unfurls quickly worldwide. The corona virus 2019 is a systemic disease which develops a prothrombotic environment, and has an extensive spectrum of clinical presentations in the gastrointestinal and hepatobiliary systems. Ischemic hepatitis (hypoxic hepatitis) is one potential mechanism behind lessened perfusion of the liver. The portal and mesenteric vein thrombosis are extremely rare complications and unusual main manifestations of COVID-19. We report the case of a patient presented acute hepatitis with portal and mesenteric vein thrombosis revealing a SARS-CoV-2 infection. In addition, we discuss the most characteristic elements of the Impact of COVID-19 on liver Injury, and the mechanisms of this damage and the formation of thrombus in portal and mesenteric vein.

## Highlights

• The COVID-19 may manifest with gastrointestinal symptoms, thromboembolic events and cardiovascular involvement.
• This COVID-19 brings pancreatitis, bowel ischemia, acute hepatitis, and gastrointestinal bleeding.
• COVID-19 has been shown to liver injury or liver dysfunction.
• The portal and mesenteric vein thrombosis are extremely rare complications and unusual main manifestations of COVID-19.

## 1 Introduction

Novel coronavirus disease 2019 (COVID-19) is a single-stranded RNA virus identified for the first time in Wuhan, China, and it unfurls quickly worldwide. This aggressive disease is majorly represented by fever, respiratory syndrome, and fatigue, similar to the influenza, and may develop into acute respiratory distress syndrome, septic shock, and respiratory or heart failure [1]. Although this contagious viral pandemic declared in MARCH 2020 has a significant predilection to affect the lower respiratory system, the COVID-19 may manifest with gastrointestinal symptoms, thromboembolic events and cardiovascular involvement. In particular, the extra-respiratory manifestations of the Covid-19 are still incomplete. The covid-19 has an extensive spectrum of clinical presentations in the gastrointestinal and hepatobiliary systems [2]. Frequently, it alters the liver as the second in second degrees leading to hepatic injury [3]. Clinical features of the gastrointestinal tract and visceral vascular system implications secondary to covid-19 vary from ordinary symptoms include anorexia and diarrhea, to intestinal infarction and extensive peritonitis [1,4].

The corona virus 2019 is a systemic disease which develops a prothrombotic environment. One-third of severe cases present thrombotic complications [5]. Herein, we report the case of a patient presented acute hepatitis with portal and mesenteric vein thrombosis revealing a SARS-CoV-2 infection. In addition, we discuss the most characteristic elements of the Impact of COVID-19 on liver Injury, and the mechanisms of this damage and the formation of thrombus in portal and mesenteric vein. This work has been reported in line with the SCARE 2020 Guideline [25].

## 2 Case presentation

A 23-year-old female patient was referred to our department for surgical management regarding Familial Adenomatous Polyposis (FAP). The initial chief complaint has been rectal bleeding lasting four years with a lower abdominal pain. Her past surgical history was unremarkable, without reference to using drugs, alcohol or tobacco. There was no history of chronic liver disease. A total colonoscopy was performed, objecting hundreds of pedunculated or sessile polyps from rectum to cecum. Histopathology was consistent with tubulovillous and villous adenomas with low-grade dysplasia. There was a history of the premature death of her mother at the age of 37 years with a history of malignant degeneration of colorectal polyps. A total proctocolectomy with a loop ileostomy was performed.

Seven days later, the reported post-operative course of our patient was marked by unexplained chest pain, dyspnea, severe asthenia, and hypotension.

On physical examination, the patient was tachycardic (135 pulse/minute), oligo-anuric with blood pressure at 80/43mmhg, oxygen saturation of 94% under an oxygen facial mask at a flow rate of 3 L/min, and respiratory rate of 39 cycles per minute. There was no jaundice. We suspected a pulmonary embolism, but the computed tomography pulmonary angiography was unremarkable. The ECG showed sinus tachycardia without reporalization disorders.

**Fig. 1:**
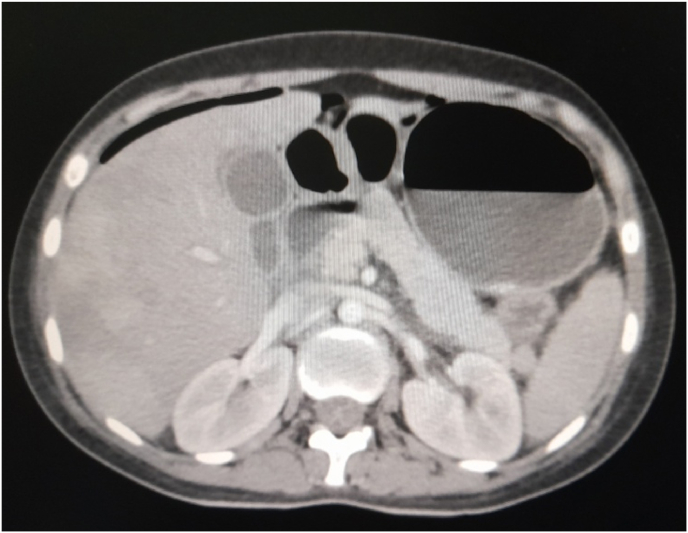
hepatic CT finding.

Abdominal examination was shown right-sided abdominal pain with a severity of 6 in 10. The chest and abdominopelvic CT-scan with *hepatic Doppler ultrasound* were performed, showing portal and mesenteric vein thrombosis with heterogeneous liver, increased in size at segment Ⅷ, presenting a mosaic appearance related to perfusion disorder (Fig. 1).

Laboratory tests included leukocytosis 14620/mm³, lymphopenia at 305/mm³, a reactive C protein at 287mg/l, AST/ALT at 327/326, LDH at 850, with positive SARS-Cov-2 RT-PCR as well as a positive SARS-CoV-2 serology (positive IgG and IgM). The serological tests for hepatitis A, B, C and E were negatives. These features strongly supported the diagnosis of acute hepatitis revealing SARS-CoV-2.

The patient was put under treatment based on Azythromycine 500mg on the first day, then 250mg for four days, Ceftriaxon 2g/d, Metronidazole 500mg/8h, High doses of methylprednisolone, Vitamin C 1g/d, zinc 45mg/d, vitamin D 25000IU/week, anticoagulants by Enoxaparin 6000UI × 2/d, optimization of electrolyte balance, and gastric protection using proton pump inhibitors 40 mg/d.

The outcome was favorable without any respiratory or hepatic impairment noted. The patient improved clinically, she was discharged within 15 days with a total resolution of her respiratory and liver abnormalities with total disappearance of the thrombus. She was seen again in consultation after 8 months, and her clinical and radiological examinations were unremarkable.

## 3 Discussion

SARS-CoV-2 infection (severe acute respiratory syndrome coronavirus 2) is a viral and emerging infection responsible of a global pandemic since its appearance in Wuhan in December 2019. This entity comes considered as a multi-systemic disease, and patients with Covid-19 present to a lesser extent cardiovascular involvement, thrombogenic ischemia in various parts of the body, and gastrointestinal symptoms [[1], [2], [3], [4]].

Common digestive tract manifestations of Covid 19 include mainly liver enzyme abnormalities, diarrhea, nausea, vomiting, and abdominal pain [6]. The most likely mechanism of this clinical presentation suggests that the angiotensin converting enzyme 2 (ACE2) receptors spread COVID-19 in the epithelium digestive tract, leading to inflammation mediation. This event brings pancreatitis, bowel ischemia, acute hepatitis, and gastrointestinal bleeding [7]. COVID-19 has been shown to liver injury or liver dysfunction [8]. Moreover, 14%–53% of COVID-19 cases present liver damage [9].

SARS-CoV2 interacts efficiently with three receptors (ACR2, TMPRSS2, and FURIN) which are expressed in liver tissue cells, and triggers direct cytopathic effect (inflammatory response) and disrupts dysregulated immune response. This viral infection leads to the apoptosis and ballooning degeneration along [10]. The pathology report of liver of patients with covid-19 shows the hepatocellular necrosis [11]. In our case, the patient was presented hepatomegaly caused by an increase in ballooned hepatocytes.

Ischemic hepatitis (hypoxic hepatitis) is one potential mechanism behind lessened perfusion of the liver, and discovers as ALT immediate and extreme elevation [12,13]. A study done by Dunn et al. [14] has stated three possible mechanisms of this injury: hepatic ischemia, venous congestion, and arterial hypoxemia. The respiratory failure caused by SARS-COV2 can reduce oxygen feeding to the liver [13,14]. According to Kulkarni et al. [15], the COVID-19 disease has impact on hepatic panel. Abnormal liver function tests were noticed in 23.1% of 20,874 covid-19 patients. Some studies have mentioned that impaired liver perfusion is secondary to microvascular thrombosis, mitochondrial dysfunction, and hepatic steatosis induced by SARS-CoV-2 that targets the liver [16]. Therefore, this can be origin of raised levels of AST which is elevated relative to ALT, showing an AST/ALT ratio >1 [17].

According to Ding et al. [18], the risk factors associated with an increased mortality rate in patients with SARS- COV-2 included high levels of conjugated bilirubin and aspartate aminotransaminase AST.

In addition, as reported by Salik et al. [19], the COVID-19 patients with liver dysfunction present elevated LDH, CK, ferritin, CRP, and procalcitonin levels compared to patients without liver damage. These elevated parameters levels have been associated with poor prognosis and a high mortality rate.

Undeniably, the drugs administered during hospitalization of covid-19 patients can lead to hepatotoxicity and liver damage. The consumption of antipyretic, antiviral drugs like remdesivir, ritonavir and lopinavir might cause abnormality in liver function. The usage of methylprednisolone to treat COVID-19 severe cases has been associated with reactivation of chronic hepatitis B virus infection, but usually with extensive use of this corticosteroid [20].

Consistently, our report of the portal and mesenteric vein thrombosis in COVID-19 case, despite have not high risk factors for thrombotic event, indicates an interesting aspect of this disease. Venous and arterial thromboembolism can occur in patients with SARS-CoV-2 viral infection because of its high thrombotic potential. Other types of coagulation disorders can be associated such as disseminated intravascular coagulation (DIC) [21].

The portal and mesenteric vein thrombosis are extremely rare complications and unusual main manifestations of COVID-19 developed within 2 weeks from admission. The mechanism of these conditions is the same which leads to the liver injury [21].

The clinical presentation of patients with thrombosis in the portal vein is heterogeneous and non-specific varied from asymptomatic patients (20%) to hepatomegaly (67%). 83% of cases present ascites, and more than 61% of patients present abdominal pain [22]. The increase in plasma D-dimer levels associated with a decrease in serum C-reactive protein CRP levels may designate the development of thrombo-embolic complications [23]. *Hepatic Doppler ultrasound* and contrast-enhanced computed topography are the best imaging techniques for the diagnosis. On CT scan, the thrombosis is manifested by bowel wall thickening, pneumatosis, and to a lesser extent portal venous gas [24].

The immediate anticoagulant therapy (Heparin and low-molecular-weight heparin) is recommended treatment to prevent progression of thrombosis of the portal vein to intestinal infarction [21]. To our best knowledge, few cases of portal vein thrombosis associated with mesenteric vein thrombosis caused by COVID-19 were reported in the literature. In this paper, we report the case of a young female COVID-19 patient who was presented vascular thrombosis associated with acute hepatitis leading to perfusion disorder.

In summary, the Coronavirus 2019 disease (COVID-19) is a relatively new condition which can act potentially on digestive tract caused serious complications such as liver damage and micro and macrovascular thrombosis. Our report clarifies the need of close monitoring of patients with Covid-19 to make the correct diagnosis and to detect early thromboembolic events, or other complications. This case, also, highlights the importance of *sanitary measures* to ensure the *safety* of all *patients* scheduled for surgery.

## Ethical approval

No ethical approval necessary.

## Sources of funding

The author(s) received no financial support for the research, authorship and/or publication of this article.

## Author contribution

All authors contributed toward data analysis, drafting and revising the paper, gave final approval of the version to be published and agree to be accountable for all aspects of the work.

**Dr Harouachi Abdelhakim:** Have written the article, have consulted the patient, prescribed all of the tests and prepared the patient for surgery and participated in the surgery.

**Pr Bouhout Tariq:** have helped writing the article, data collection.

**Pr Hanane Hadj Kacem:** have supervised the writing of the paper.

**Pr Serji Badr**: have supervised the writing of the paper.

**Pr Berkhli Hayat:** have supervised the writing of the paper.

**Pr Madani Hamid**: have supervised the writing of the paper.

**Pr El Harroudi Tijani** (oncology surgery professor): Writing, review and editing of the manuscript, and had been the leader surgeon of the case.

## Registration of research studies

Our paper is a case report; no registration was done for it.

## Patient consent

Written informed consent was obtained from the patient for publication of this case report and accompanying images. A copy of the written consent is available for review by the Editor-in-Chief of this journal on request.

## Guarantor

Harouachi Abdelhakim.

## Provenance and peer-review

Not commissioned, externally peer reviewed.

## Funding information

This research has not been supported by any private or corporate financial institutions nor has any grant been received for this study.

## Declaration of competing interest

The authors declare that they have no conflict of interest.
